# Identification and Genetic Characterization of *Ralstonia solanacearum* Species Complex Isolates from *Cucurbita maxima* in China

**DOI:** 10.3389/fpls.2017.01794

**Published:** 2017-10-18

**Authors:** Xiaoman She, Lin Yu, Guobing Lan, Yafei Tang, Zifu He

**Affiliations:** ^1^Plant Protection Research Institute Guangdong Academy of Agricultural Sciences, Guangzhou, China; ^2^Guangdong Provincial Key Laboratory of High Technology for Plant Protection, Guangzhou, China

**Keywords:** *Ralstonia solanacearum*, identification, bacterial wilt, *Cucurbita maxima*, phylotype I, sequevar

## Abstract

*Ralstonia solanacearum* species complex is a devastating phytopathogen with an unusually wide host range, and new host plants are continuously being discovered. In June 2016, a new bacterial wilt on *Cucurbita maxima* was observed in Guangdong province, China. Initially, in the adult plant stage, several leaves of each plant withered suddenly and drooped; the plant then wilted completely, and the color of their vasculature changed to dark brown, ultimately causing the entire plant to die. Creamy-whitish bacterial masses were observed to ooze from crosscut stems of these diseased plants. To develop control strategies for *C. maxima* bacterial wilt, the causative pathogenic isolates were identified and characterized. Twenty-four bacterial isolates were obtained from diseased *C. maxima* plants, and 16S rRNA gene sequencing and pathogenicity analysis results indicated that the pathogen of *C. maxima* bacterial wilt was *Ralstonia solanacearum*. The results from DNA-based analysis, host range determination and bacteriological identification confirmed that the 24 isolates belonged to *R. solanacearum* phylotype I, race 1, and eight of these isolates belonged to biovar 3, while 16 belonged to biovar 4. Based on the results of partial *egl* gene sequence analysis, the 24 isolates clustered into three *egl-* sequence type groups, sequevars 17, 45, and 56. Sequevar 56 is a new sequevar which is described for the first time in this paper. An assessment of the resistance of 21 pumpkin cultivars revealed that *C. moschata* cv. Xiangyu1 is resistant to strain RS378, *C. moschata* cv. Xiangmi is moderately resistant to strain RS378, and 19 other pumpkin cultivars, including four *C. maxima* cultivars and 15 *C. moschata* cultivars, are susceptible to strain RS378. To the best of our knowledge, this is the first report of *C. maxima* bacterial wilt caused by *R. solanacearum* race 1 in the world. Our results provide valuable information for the further development of control strategies for *C. maxima* wilt disease.

## Introduction

Bacterial wilt, which is caused by the *Ralstonia solanacearum* species complex (RSSC), is one of the most important and devastating plant diseases in the tropics, subtropics and other warm temperate areas of the world (Hayward, 1991). *Ralstonia solanacearum* has a very wide plant host range—more than 200 plant species belonging to over 50 botanical families (Denny, 2006). Traditionally, *R. solanacearum* has been divided into five races based on host range (Buddenhagen et al., 1962; Pegg and Moffett, 1971; He et al., 1983), and six biovars have been described based on their ability to oxidize three disaccharides and three hexose alcohols (Hayward, 1964, 1991, 1994; He et al., 1983).

Recently, molecular methods have resulted in a greater understanding of the diversity of *R. solanacearum*. Cook et al. (1989) and Cook and Sequeira (1994) distinguished 46 multilocus genotypes of *R. solanacearum* by restriction fragment length polymorphism (RFLP) analysis and clustered *R. solanacearum* strains into two divisions, division I and division II. Division I includes biovar 3, 4, and 5 strains originating from Australia and Asia, and division II includes biovar 1, 2, and 2T strains originating from the Americas. Two more groups of strains were identified by fingerprinting analyses using amplified fragment length polymorphism (AFLP) and polymerase chain reaction-RFLP (PCR-RFLP) techniques on targeting the HRP cluster gene (Poussier et al., 2000a), 16S rRNA gene (Li et al., 1993; Taghavi et al., 1996; Poussier et al., 2000a), intergenic spacer region between the 16S and 23S rRNA genes (ITS), and endoglucanase and *hrpB* genes (Fegan et al., 1998; Poussier et al., 2000b). One group includes biovar 1 and 2T strains from Africa, and another group includes strains belonging to biovars 1, 2, and 2T from Indonesia as well as two closely related strains of *Ralstonia syzygii*, a pathogen from cloves, and the bacterial agent of blood disease of banana (BDB) (Taghavi et al., 1996).

*Ralstonia solanacearum* is considered a “species complex” due to the significant genetic diversity that exists within the species, which was identified by Fegan and Prior (2005), who proposed a new hierarchical classification scheme for *R. solanacearum*. The *R. solanacearum* species complex comprises species, phylotypes, sequevars, and clones in this new hierarchical classification (Fegan and Prior, 2005). The phylotypes are defined into monophyletic clusters by phylogenetic analysis of the ITS region, *hrpB* gene and *egl* gene. Phylotypes I and II are equivalent to divisions I and II (Cook and Sequeira, 1994). Phylotype III contains strains from Africa. Phylotype IV contains strains from Indonesia as well as *R. syzygii* and BDB. These phylotypes are identified via multiplex PCR with *R. solanacearum* species-specific primers 759/760 and phylotype-specific primers (Nmult:21:1F, Nmult:21:2F, Nmult:23:AF, Nmult:22:InF, and Nmult:22:RR). Strains of *R. solanacearum, R. syzygii* and BDB generate a species-specific 280-bp fragment with primers 759/760, and strains in phylotypes I to IV generate fragments of 144, 372, 91, and 213 bp, respectively, with the phylotype-specific primers. Phylotypes are subdivided into sequevars based on the analysis of partial *egl* gene sequences and into clones by genetic fingerprinting techniques such as pulsed field gel electrophoresis (PFGE) and repetitive-sequence-based polymerase chain reaction (rep-PCR) (Fegan and Prior, 2005). To date, 55 sequevars of *R. solanacearum* have been identified (Li et al., 2016; Liu et al., 2017). Furthermore, based on a polyphasic taxonomic approach that emphasizes DNA-DNA hybridization analysis, the *R. solanacearum* species complex comprises three genospecies (Safni et al., 2014). Safni et al. suggested that *R. pseudosolanacearum* sp. nov. corresponds to phylotypes I and III, *R. solanacearum* corresponds to phylotype II, and *R. syzygii* corresponds to phylotype IV (Safni et al., 2014). According to an analysis of genotypic and phenotypic data and genomic sequences, Safni et al. also suggested that *R. syzygii* be divided into three subspecies: *R. syzygii* subsp. *syzygii* subsp. nov., which corresponds to the current *R. syzygii* strains; *R. syzygii* subsp. *indonesiensis* subsp. nov., which corresponds to the current *R. solanacearum* phylotype IV strains; and *R. syzygii* subsp. *celebesensis* subsp. nov., which corresponds to the BDB strains (Safni et al., 2014). In 2016, using a combination of phenotypic analyses, whole-genome comparisons and proteomic profiling, Prior et al. suggested that the *R. solanacearum* species complex be divided into three species: the first species includes phylotypes I and III, the second species corresponds to phylotype II, and the third species comprises the phylotype IV strains, *R. syzygii*, and the BDB strains (Prior et al., 2016).

Bacterial wilt is one of the most important crop diseases, especially in southern China, and it was first observed in peanuts in the early 1930s (Ma and Gao, 1956). Later, this disease was reported in sweet potatoes (*Ipomoea batatas* (L.) Lam) (Zheng and Fan, 1962), horsetail beefwood (*Casuarina equisetifolia* L.), mulberry (*Morus alba* L.) and eucalyptus (*Eucalyptus* spp.) (He et al., 1983; He, 1985). Over time, even more hosts were found to be susceptible to *R. solanacearum* infection. In China, more than 28 host plants of *R. solanacearum* have been reported within the past decade (He et al., 2008; Liu et al., 2009; Wang et al., 2009; Xu et al., 2009; She et al., 2012, 2013), and new host plants are continuously being discovered. In June 2016, *Cucurbita maxima* bacterial wilt was first observed in a field in Guangzhou City, Guangdong province, China. The plant symptoms of *C. maxima* bacterial wilt are similar to those of other host plants bacterial wilt. We obtained 24 bacterial isolates from diseased plants of *C. maxima* and conducted the present study to identify the causal agent of *C. maxima* bacterial wilt.

## Materials and methods

### Symptoms

**Figure 1:**
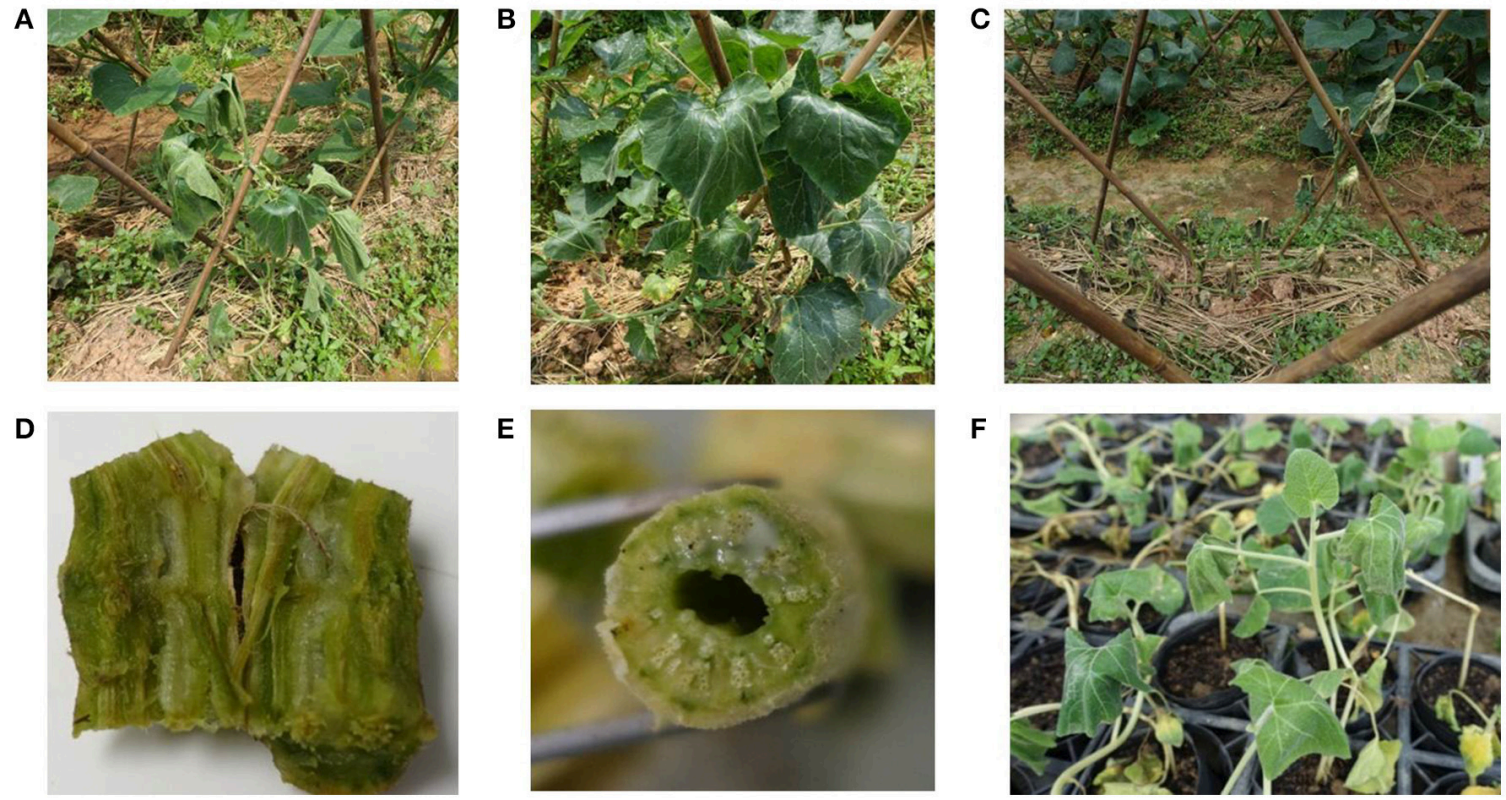
Top leaf stunting **(A)**, wilting plants **(B)**, and plant death **(C)** of *C. maxima* in fields; **(D)** vascular discoloration of a *C. maxima* stem infected with *R. solanacearum*; **(E)** bacteria oozing from the crosscut site of a *C. maxima* stem; and **(F)** inoculated plants showing wilt symptoms in a greenhouse.

Disease development in *C. maxima* begins with drooping of the half side of the top leaf (Figure 1A) and continues with lower leaf wilt and droop, whole plant wilt (Figure 1B), changes in the color of the vasculature to dark brown (Figure 1D), and death of the plant (Figure 1C). These symptoms are similar to those caused by *R. solanacearum* in other host plants. Bacterial oozing from the stem crosscut sites of plants infected with *R. solanacearum* was observed (Figure 1E).

### Pathogen isolation

To isolate the putative causative agent from diseased plants, 24 wilted plants of *C. maxima* were collected from fields in the Baiyun district in Guangzhou City, Guangdong province, China. An approximately 1.5 × 0.5 cm section of the basal stem from each diseased plant was excised, surface-sterilized with 70% (v/v) ethanol for 30 s and 0.5% (v/v) sodium hypochlorite for 30 s, and then rinsed with sterile water 4–6 times. An approximately 0.5 × 0.5 mm section of vascular tissue from the surface-sterilized stem section was mashed and macerated in 50 μl of sterile distilled water and left to stand for 2–3 min. These suspensions were then streaked on 2,3,5-triphenyltetrazolium chloride (TZC) medium plates (Kelman, 1954) and incubated at 30°C for 2 days. Large, irregular, round, fluidal and white colonies with pink center pre-dominated the TZC medium plates. One isolate was obtained from each plate after two successive single-colony isolations. In all, 24 isolates were stored in 15% (w/v) glycerol at −80°C for further use.

### Pathogenicity analysis

Virulence tests were performed on four- to six-leaf stage plants of *C. maxima*, tomato (*Lycopersicon esculentum*, cv. Dongqie), pepper (*Capsicum annuum*, cv. Yuejiao No. 15), eggplant (*Solanum melongena*, cv. Yuefeng), tobacco (*Nicotiana tabacum*), banana (*Musa nana*, cv. Dafeng No. 1) and ginger (*Zingiber officinale*). Fifteen plants of each host were inoculated with each isolate. Six of the seven hosts (excluding ginger), were inoculated by injuring the roots and soaking them in a bacterial suspension (1 × 10^8^ cfu/ml) for 20 min. The roots of 15 plants of each host were also injured and soaked in fluid nutrient medium as negative controls. Fifteen ginger plants were inoculated by injection with 200 μl of a bacterial suspension (1 × 10^8^ cfu/ml) in their stem bases. Fifteen additional ginger plants were injected with 200 μl of fluid nutrient medium as negative controls. After inoculation, the plants were incubated at 28–30°C and 75–80% relative humidity. Disease incidence (DI) was monitored every week for 5 weeks. Plants with visible symptoms (wilted leaves) were recorded as diseased plants. The disease incidence was calculated as DI (%) = 100 × number of disease plants/15 inoculated plants. The experiment was repeated three times. The bacterial pathogen was re-isolated from inoculated *C. maxima* plants exhibiting wilt symptoms.

### DNA preparation

DNA was extracted from each isolate using an EasyPure® Genomic DNA kit (TransBionovo Co., Ltd., China) according to the manufacturer's instructions, and the quality and quantity of the DNA samples were checked by measuring their A260/A280 ratios using a NanoDrop One Microvolume UV-Vis Spectrophotometer (Instrument operating software version 1.2.0, Thermo Fisher Scientific, Inc.). The DNA samples were stored at −20°C until use.

### DNA sequencing of the 16S rRNA gene

The DNA samples were used as templates for PCR amplification of the 16S rRNA gene using the universal primers 27f/1491r (Woese et al., 1983). Approximately 50 ng of DNA template was added to 12.5 μl of Premix Taq™ (0.625 U of TaKaRa Taq, 0.2 mM dNTP mixture, PCR buffer, 10 mM Tris-HCl, pH 8.3, 50 mM KCl, and 1.5 mM MgCl_2_) (TaKaRa Biotechnology Co., Ltd, China), 10 pmoles of the primers 27f and 1491r (Woese et al., 1983) and ddH_2_O to a final volume of 25 μl. The PCR program consisted of an initial denaturation at 96°C for 5 min, followed by 35 cycles of 94°C for 1 min, 48°C for 1 min and 72°C for 3 min, and a final extension at 72°C for 10 min in a Mastercycler® Gradient Thermal Cycler (Eppendorf, Germany). The PCR product for each strain was subjected to electrophoresis on a 1% agarose gel at 100 V/cm for 40 min, which was then stained with DNA Green (TIANDZ, China) and visualized using a UV-transilluminator.

Each resulting amplicon was ligated into the vector pMD-20T (TaKaRa Biotechnology Co., Ltd, China). The 16S rRNA fragment-containing recombinant plasmids were transformed into *Escherichia coli* strain JM109. Three clones were randomly picked from each transformation and sequenced in both directions using primer walking (Invitrogen Life Technologies China Co., Ltd, China). The pathogens were identified by blasting the obtained 16S rRNA gene sequences against the NCBI database.

### Biovar determination

Biovars of the 24 isolates were determined as described by Hayward (1964). Briefly, 10 μl of a cultured bacterial suspension (1 × 10^8^ cfu/ml) were stabbed into slants of Hayward's medium containing 1% filter-sterilized D-(+)-cellobiose, lactose, maltose, sorbitol, mannitol or dulcitol in glass tubes. All of the inoculated tubes were incubated at 30°C for 3 weeks. Each treatment was repeated three times. Liquid culture media inoculation was used as a negative control. Color changes of the Hayward's medium slants from green to yellow were recorded as positive.

### Phylotype-specific multiplex PCR (Pmx-PCR) amplification

The strain phylotypes was determined utilizing a phylotype-specific multiplex PCR (Pmx-PCR) assay following a previously described method (Fegan and Prior, 2005; Prior and Fegan, 2005). Approximately 50 ng of DNA template was added to 12.5 μl of Premix Taq™ (0.625 U of TaKaRa Taq, 0.2 Mm dNTP Mixture, PCR buffer, 10 mM Tris-HCl, pH 8.3, 50 mM KCl, and 1.5 mM MgCl_2_) (Takara Co.); 6 pmoles of the primers Nmult:21:1F, Nmult:21:2F, and Nmult:22:InF; 18 pmoles of the primer Nmult:23:AF; 4 pmoles of the primers 759 and 760 (Opina et al., 1997; Xu et al., 2009); and ddH_2_O to a final volume of 25 μl. The PCR program included an initial denaturation at 96°C for 5 min, followed by 30 cycles of 94°C for 15 s, 50°C for 30 s and 72°C for 30 s, and a final extension at 72°C for 10 min in a Mastercycler® Gradient Thermal Cycler (Eppendorf, Germany). Eight microliters of PCR product for each strain was subjected to electrophoresis on a 2% agarose gel at a voltage of 100 V/cm for 40 min, which was then stained with DNA Green (TIANDZ, China) and visualized using a UV-transilluminator.

### Partial sequencing of the endoglucanase (*egl*) and *hrpB* genes

PCR amplification of the 1,360-bp region of the *egl* gene was performed using the primers eglF (5′-TCTCCATTTTTCCATTTCGTCATG-3′) and eglR (5′-ATGCCATC CGCCACGGACCCGGC-3′). Approximately 50 ng of DNA template was added to 25 μl of Premix Taq™, 0.5 μM of each primer, 2 μl of dimethyl sulfoxide and ddH_2_O to a final volume of 50 μl. The PCR program included an initial denaturation at 96°C for 9 min, followed by 35 cycles of 94°C for 1 min, 62°C for 1 min and 72°C for 2.5 min, and a final extension at 72°C for 10 min in a Mastercycler® Gradient Thermal Cycler. PCR amplification of the 1,434-bp region of the *hrpB* gene was performed using the primers hrpBF (5′-TGACGCTTCAGGAGCATTGCC-3′) and hrpBR (5′-AGATGGGAAGGGAGAGGACC-3′). Approximately 50 ng of DNA template was added to 25 μl of Premix Taq™, 0.5 μM of each primer, 2 μl of dimethyl sulfoxide and ddH_2_O to a final volume of 50 μl. The PCR program included an initial denaturation at 96°C for 5 min, followed by 35 cycles of 94°C for 1 min, 62°C for 1 min and 72°C for 3 min, and a final extension at 72°C for 10 min using a Mastercycler® Gradient Thermal Cycler (Eppendorf, Germany). Six microliters of PCR product from each strain was subjected to electrophoresis on a 1% agarose gel at a voltage of 120 V/cm for 25 min, which was then stained with DNA Green and visualized using a UV-transilluminator. The PCR products were purified and sequenced by Shanghai Sangon Biological Engineering Technology & Service CO., Ltd. The PCR primers were also used as sequencing primers.

### Sequence analysis

Twenty-three sequences of the *egl* gene and 21 sequences of the *hrpB* gene were selected for analysis (Table 1). The sequences were edited with BioEdit 7.0.5.1 (Hall, 1999) and aligned using Clustal W in DNASTAR MegAlign software, version 5.01 (DNASTAR Inc., Madison, WI, USA). Phylogenetic trees were constructed using the neighbor-joining (NJ) and maximum-likelihood (ML) methods embedded in MEGA version 6.0 (Tamura et al., 2013). Bootstrapping was performed with 100 replicates for the ML tree and 1,000 replicates for the NJ tree. All of the *egl* and *hrpB* sequences from the *R. solanacearum* strains isolated in this study were deposited into GenBank (Table 1).

**Table 1:** Strains used in this study.

**Isolates**	**Race**	**Biovar**	**Phylotype**	**GenBank accession no**.
RS345	1	3	I/17	KY594789
RS346	1	3	I/17	KY594790
RS347	1	3	I/17	KY594791
RS348	1	3	I/17	KY594792
RS353	1	4	I/56	KY594793
RS355	1	4	I/45	KY594794
RS356	1	4	I/45	KY594795
RS357	1	4	I/45	KY594796
RS362	1	3	I/56	KY594797
RS364	1	3	I/45	KY594798
RS365	1	3	I/45	KY594799
RS366	1	3	I/45	KY594800
RS371	1	4	I/56	KY594801
RS372	1	4	I/45	KY594802
RS373	1	4	I/45	KY594803
RS374	1	4	I/45	KY594804
RS375	1	4	I/45	KY594805
RS376	1	4	I/45	KY594806
RS377	1	4	I/45	KY594807
RS378	1	4	I/45	KY594808
RS379	1	4	I/45	KY594809
RS380	1	4	I/45	KY594810
RS381	1	4	I/45	KY594811
RS382	1	4	I/45	KY594812
**Reference strains for** ***egl***				
**Strains**	**host**	**Phylotype**	**GenBank accession no**.	**References**
CFBP3059	Eggplant	III/23	AF295270	Castillo and Greenberg, 2007
MAFF301552	Tomato	IV/8	GU295042	
DGBBC1125	Potato	III/43	GU295008	
CMR33	Tomato	III/20	GU295007	
CFBP1184	*Musa sp*	II/4	GU294935	
UW28	Potato	II/3	GU295054	
CFBP4787	Potato	II/1	GU294986	
CFBP1410	*Bnana plantain*	II/2	GU294936	
R288	*Morus alba*	I/12	GQ907153	Fegan and Prior, 2005
R28	*Syzygium aromaticum*	IV/9	DQ011552	Fegan and Prior, 2006
Pss4	Tomato	I/15	EU407264	Lin et al., 2014
CMR134	Hucklererry	I/13	EF439740	Gabriel et al., 2009
CIP365	Potato	I/45	GQ907151	Prior and Fegan, 2005
Psi7	Tomato	IV/10	EF371804	
ACH732	Tomato	IV/11	GQ907150	
CFBP6941	Tomato	III/29	EF439743	Wicker et al., 2012
Pe3	Pepper	I/44	FJ561155	Xu et al., 2009
O3	Olive tree	I/44	FJ561069	
Tm1301	Tomato	I/44	FJ561087	
Tm2	Tomato	I/14	FJ561134	
M4	Mulberry	I/12	FJ561107	
Pe11	Pepper	I/17	FJ561084	
PSS219	Tomato	I/34	FJ561091	
**Reference strains for** ***hrpB***				
**Strain**	**Host**		**GenBank accession no**.	**References**
Aoyu	/		FJ561168	Xu et al., Unpublished
CFBP734	Potato		AF295627	Poussier et al., 2000a
CFBP2047	Tomato		AF295615	
CFBP2958	Tomato		AF295619	
CFBP2972	Potato		AF295617	
GMI1000	Tomato		AF295604	
JT516	Potato		AF295611	
NCPPB3987	Potato		AF295614	
R292	*Morus alba*		AF295608	
CFBP3059	Eggplant		DQ657471	Castillo and Greenberg, 2007,
MAFF301558	Potato		AB508398	
NCPPB332	Potato		DQ657473	
M4	Mulberry		FJ561181	Xu et al., 2009
Pe11	Pepper		FJ561191	
Po2	Potato		FJ561199	
Po10	Potato		FJ561195	
Tb9	Tobacco		FJ561223	
Tm1	Tomato		FJ561224	
Tm10	Tomato		FJ561225	
Tm11	Tomato		FJ561226	
Tm82	Tomato		FJ561234	

### Evaluation of the resistance of pumpkin cultivars to *R. solanacearum*

Twenty-one pumpkin cultivars, including four cultivars of *C. maxima* and 17 cultivars of *C. moschata*, were collected and used for resistance evaluation. Plants with four true leaves were inoculated by injuring the roots and soaking them in a bacterial suspension (1 × 10^8^ cfu/ml of strain RS378) for 20 min. Inoculated plants were transplanted into plastic pots (9 cm in diameter) containing horticultural soil. This experiment included four plot experiments with 30 plants each. The DI was monitored every week for 5 weeks after inoculation. Plants with visible symptoms (leaf wilt) were recorded as diseased plants. The disease incidence was calculated as DI (%) = 100 × number of disease plants/total number of inoculated plants in each plot experiment (30). The DI ranged from 0 (no disease) to 100% (dead). Analysis of variance (ANOVA) was used to analyze the disease incidence data from each assessment using a Data Processing System (DPS) based on Duncan's new multiple range method (Hanzhou RuiFeng Information Technology Co., Ltd).

The resistance of the 21 pumpkin cultivars to strain RS378 was evaluated for 5 weeks after inoculation and categorized as follows: DI value of (i) 0–20.0%, highly resistant (HR); (ii) 20.1–40.0%, resistant (R); (iii) 40.1–60.0%, moderately resistant (MR); (iv) 60.1–80.0%, susceptible (S); and (v) 80.1–100%, highly susceptible (HS).

## Results

### Bacterial isolation and biovar determination

**Figure 2:**
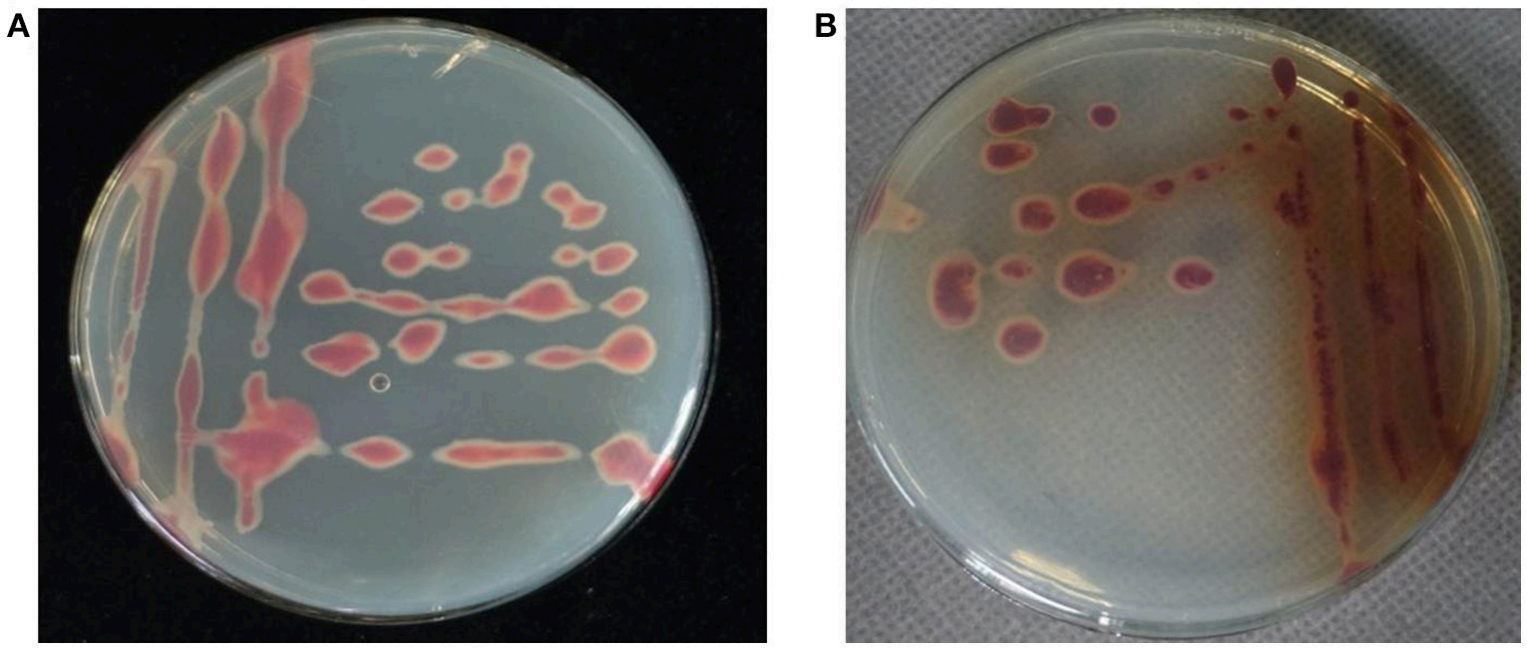
Morphology of *R. solanacearum* biovar 3 isolate RS348 **(A)** and biovar 4 isolate RS371 **(B)** on TZC medium plates.

Pure bacterial cultures were isolated on TZC medium plates from the basal stem tissues of *C. maxima* plants showing wilt symptoms. A total of 24 isolates were obtained. All of the isolates had irregular, round, fluidal, white colonies with pink center that predominated on TZC medium plates. However, 16 of the 24 isolates secreted a brown pigment after incubation at 30°C for 2 days (Figure 2). The phenotypic characterization for biovar determination revealed a predominance of biovar 4 strains over biovar 3 from *C. maxima*. Of the 24 tested isolates, eight isolates utilized maltose, lactose, D-(+)-cellobiose, mannitol, sorbitol and dulcitol and thus belonged to biovar 3, and 16 isolates secreted brown pigment on TZC medium and utilized mannitol, sorbitol and dulcitol but not maltose, lactose and D-(+)-cellobiose and thus belonged to biovar 4 (Table 1). No other biovars were found.

### Pathogen identification

Using the universal PCR primers 27f and 1491r, each of the 24 isolates generated a 1,423-bp 16S rRNA gene fragment. BLAST analysis of these 16S rRNA gene sequences revealed that they shared 100% identity with *R. solanacearum* strain GMI1000 (GenBank accession no. AL646052). The 16S rRNA gene sequences of these isolates were deposited in GenBank under accession numbers KY594769 to KY594788 and KX363800 to KX363803. These results established that the isolates from the diseased *C. maxima* plants exhibiting wilt symptoms in Guangdong, China, were *R. solanacearum*.

### Pathogenicity test

One or two of the youngest leaves of the *C. maxima* plants inoculated with the *R. solanacearum* isolates began to successively exhibit wilting at 5 days post inoculation (dpi) (Figure 1F). The initial symptom in diseased plants was apical leave wilting during the day that recovered at night. Subsequently, the wilted leaves failed to recover at night, and the whole plant withered 3 days later. No symptoms were observed in the control plants. Pathogens with the same morphology on TZC medium plates as that of the bacterial isolates mentioned above were also re-isolated from the inoculated plants with wilt symptoms. Based on these results, the pathogen causing wilt in *C. maxima* was confirmed to be *R. solanacearum*.

Inoculated plants of tomato, eggplant and pepper began to wilt at 5 dpi. The number of wilted plants stabilized at 35 dpi. Statistical analysis revealed that all of the 24 isolates were pathogenic to tomato and eggplant, with disease incidences of 66.7–100%; to pepper, with disease incidences of 26.6–86.7%; and to tobacco and ginger, with disease incidences of 0–26.67%. These isolates were not pathogenic to banana at 35 dpi (Table 2).

**Table 2:** Pathogenicity of bacterial strains isolated from *C. maxima*.

**Isolates**	**Average incidence (%)** ± **SD value**^a^						
	***C. maxima***	***S. lycopersicum* (Dongqie)**	***S. melongena* (Yuefeng)**	***C. annuum* (YuejiaoNo.1)**	***N. tabacum***	***Z. officinale***	***M. nana* (Dafeng no.1)**
RS345	97.78 ± 3.85	86.67 ± 6.67	93.33 ± 6.67	86.67 ± 6.67	6.67 ± 0	13.33 ± 6.67	0.00
RS346	97.78 ± 3.85	93.33 ± 6.67	80.00 ± 6.67	82.22 ± 16.78	6.67 ± 6.67	20.00 ± 6.67	0.00
RS347	100.00 ± 0	100.00 ± 0	73.33 ± 6.67	86.67 ± 13.33	13.33 ± 6.67	26.67 ± 6.67	0.00
RS348	97.78 ± 3.85	86.67 ± 11.55	86.67 ± 13.33	80.00 ± 20.00	6.67 ± 0	20.00 ± 6.67	0.00
RS353	97.78 ± 3.85	93.33 ± 11.55	66.67 ± 13.33	66.67 ± 13.33	13.33 ± 6.67	0.00	0.00
RS355	95.56 ± 3.85	88.89 ± 10.18	80.00 ± 20.00	46.67 ± 20.00	6.67 ± 6.67	0.00	0.00
RS356	100.00 ± 0	93.33 ± 6.67	82.22 ± 7.70	53.33 ± 6.67	0.00	0.00	0.00
RS357	100.00 ± 0	84.44 ± 10.18	75.56 ± 16.78	60.00 ± 11.55	6.67 ± 6.67	0.00	0.00
RS362	100.00 ± 0	86.67 ± 13.33	86.67 ± 13.33	86.67 ± 13.33	13.33 ± 6.67	0.00	0.00
RS364	100.00 ± 0	86.67 ± 0	95.56 ± 7.70	80.00 ± 11.55	6.67 ± 0	6.67 ± 6.67	0.00
RS365	100.00 ± 0	82.22 ± 16.78	80.00 ± 17.64	80.00 ± 17.64	26.67 ± 6.67	13.33 ± 0	0.00
RS366	95.56 ± 3.85	93.33 ± 6.67	86.67 ± 13.33	66.67 ± 6.67	13.33 ± 6.67	0.00	0.00
RS371	100.00 ± 0	86.67 ± 6.67	73.33 ± 6.67	44.44 ± 7.70	10.00 ± 3.85	0.00	0.00
RS372	100.00 ± 0	73.33 ± 6.67	66.67 ± 6.67	53.33 ± 6.67	13.33 ± 3.85	0.00	0.00
RS373	100.00 ± 0	93.33 ± 11.55	80.00 ± 13.33	53.33 ± 6.67	6.67 ± 6.67	0.00	0.00
RS374	100.00 ± 0	86.67 ± 17.64	73.33 ± 17.64	26.67 ± 11.55	6.67 ± 0	0.00	0.00
RS375	100.00 ± 0	86.67 ± 13.33	93.33 ± 6.67	46.67 ± 6.67	13.33 ± 6.67	0.00	0.00
RS376	100.00 ± 0	80.00 ± 11.55	77.78 ± 10.18	26.67 ± 6.67	6.67 ± 6.67	0.00	0.00
RS377	100.00 ± 0	86.67 ± 13.33	93.33 ± 6.67	46.67 ± 6.67	6.67 ± 6.67	0.00	0.00
RS378	100.00 ± 0	93.33 ± 6.67	66.67 ± 6.67	33.33 ± 6.67	20.00 ± 6.67	0.00	0.00
RS379	100.00 ± 0	80.00 ± 13.33	80.00 ± 0	26.67 ± 6.67	6.67 ± 6.67	0.00	0.00
RS380	100.00 ± 0	93.33 ± 6.67	86.67 ± 6.67	46.67 ± 6.67	0.00	0.00	0.00
RS381	95.56 ± 3.85	86.67 ± 11.55	86.67 ± 17.64	33.33 ± 11.55	6.67 ± 6.67	0.00	0.00
RS382	95.56 ± 3.85	93.33 ± 6.67	93.33 ± 6.67	40.00 ± 11.55	6.67 ± 6.67	0.00	0.00
RS382	95.56 ± 3.85	93.33 ± 6.67	93.33 ± 6.67	40.00 ± 11.55	6.67 ± 6.67	0.00	0.00

a *Disease incidence (DI) of diseased plants were assessed at 5 weeks after inoculation, calculated as DI = 100 × number of disease plants/15 inoculated plants*.

### Phylotyping and phylogenetic analysis

Using the Pmx-PCR protocol, each of the 24 isolates generated the expected 280-bp species-complex-specific fragment and a 144-bp fragment specific to phylotype I of *R. solanacearum*, suggesting that all of the isolates from *C. maxima* that cause wilting disease should be phylotype I strains.

**Figure 3:**
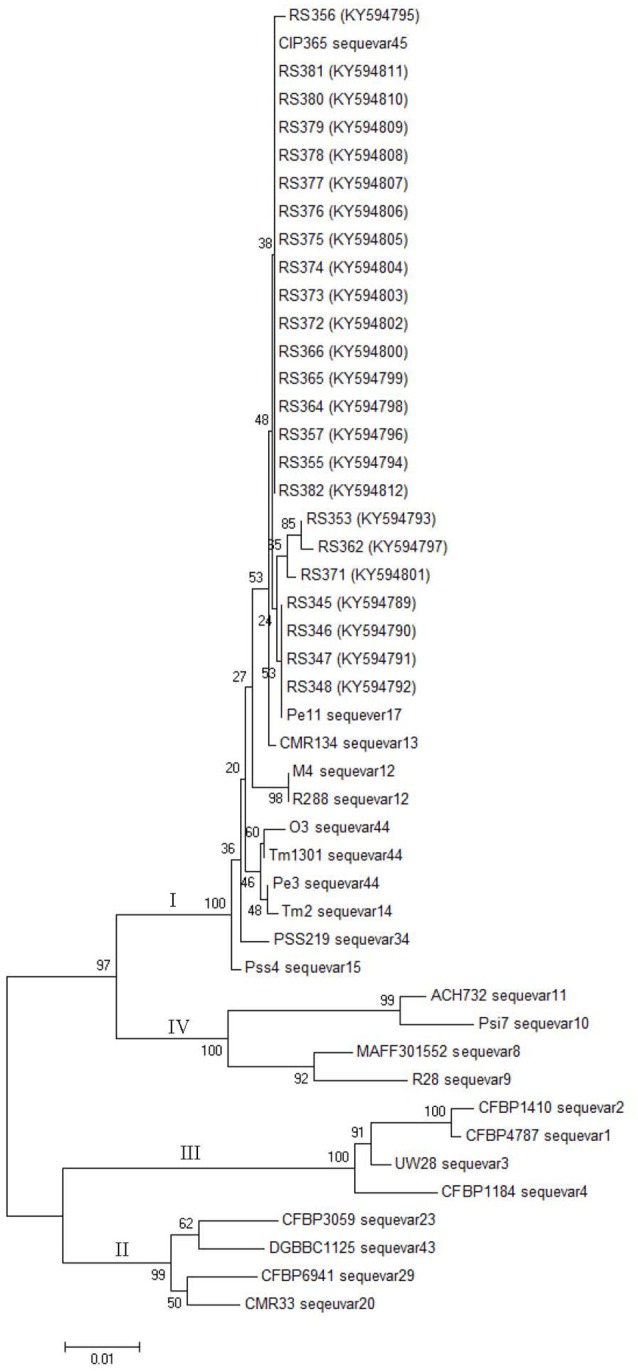
Phylogenetic analysis of 24 isolates from *C. maxima* and 23 related strains of *R. solanacearum*. The phylogenetic tree of *egl* gene partial sequences was constructed with MEGA 6.0 using the neighbor-joining method. The numbers at the tree branch points indicate the percent bootstrap support for 1,000 iterations.

**Figure 4:**
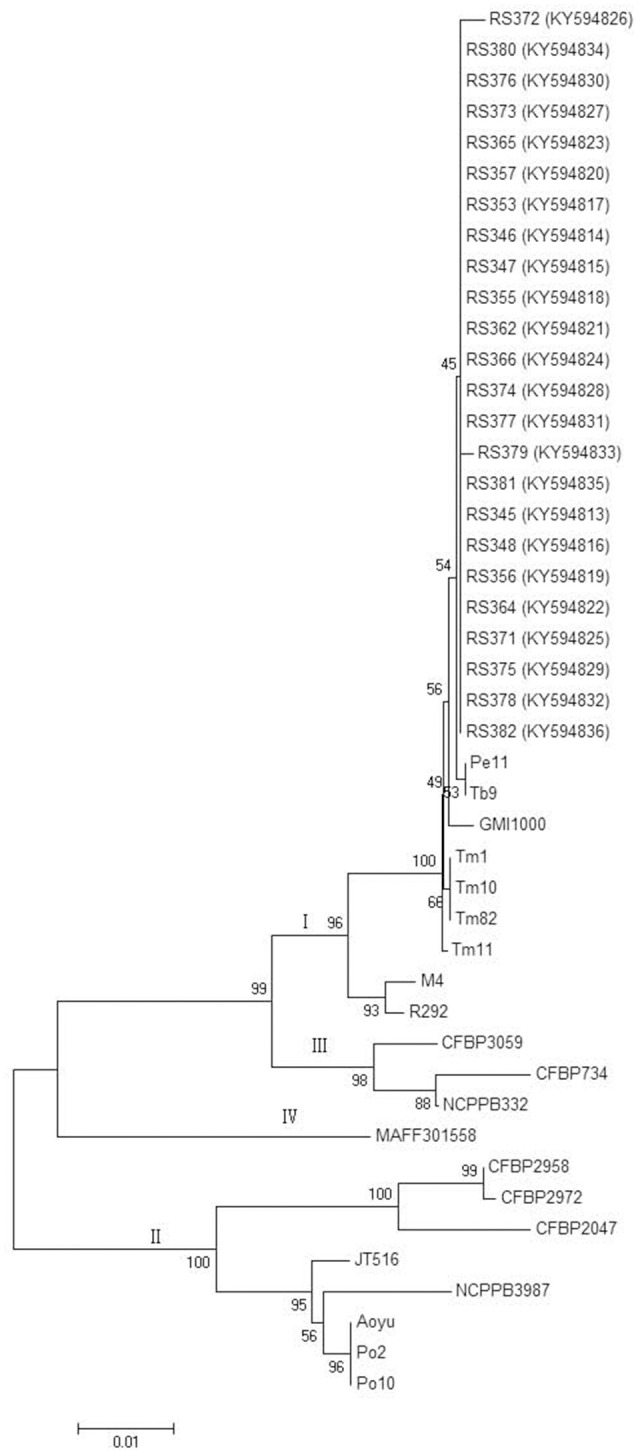
Phylogenetic analysis of 24 isolates from *C. maxima* and 21 related strains of *R. solanacearum*. The phylogenetic tree of *hrpB* gene partial sequences was constructed with MEGA 6.0 using the neighbor-joining method. The numbers at the tree branch points indicate the percent bootstrap support for 1,000 iterations.

Partial sequences of the *egl* and *hrpB* genes from these 24 isolates were analyzed. The *egl* gene sequences from the 24 isolates shared identities of 99–100% with each other. The *hrpB* gene sequences from the 24 isolates shared identities of 99.1–100% with each other. These sequences were deposited in GenBank under the accession numbers KY594789 to KY594812 for the *egl* gene and KY594813 to KY594836 for the *hrpB* gene. Phylogenetic trees were constructed using *egl* gene sequences of the 24 isolates from *C. maxima* and 23 reference strains and *hrpB* gene sequences of the 24 isolates from *C. maxima* and 21 reference strains of *R. solanacearum*, which were retrieved from GenBank. The reference strain sequences were added to the phylogenetic tree to position the strains in this study within the known phylogenetic structure (Liu et al., 2017). Two phylogenetic methods (NJ and ML) yielded similar results, but only the NJ tree is displayed for discussion. Based on the *egl* and *hrpB* trees, all of the 24 isolates in this study were classified into phylotype I, consistent with results from Pmx-PCR. The branching patterns in the *egl* and *hrpB* trees for phylotype I showed a split with 100 and 96% bootstrap values, respectively. In the *egl* tree, the 24 isolates clustered into three major groups, A, B and C. Group A, which includes 17 isolates, represented sequevar 45. Group B, which contains three isolates, was not identical to any previously designated sequevar and was thus defined as sequevar 56, a new sequevar. Group C, which included four isolates, represented sequevar 17 (Figure 3). Conversely, in the *hrpB* tree, the 24 isolates clustered into one group (Figure 4).

### Resistance assessment of pumpkin cultivars

Inoculated pumpkin plants began to wilt at 5 dpi. The number of diseased plants stabilized at 35 dpi. Statistical analysis revealed that none of the 21 pumpkin cultivars was immune to *R. solanacearum* strain RS378. The disease incidences for the 21 cultivars ranged from 38.63 to 94.92%. Xiangyu1, a *C. moschata* cultivar with a DI value of 38.63%, was resistant to strain RS378. Xiangmi, a *C. moschata* cultivar with a DI value of 57.88%, was moderately resistant to strain RS378. Seven *C. moschata* cultivars, including Xiangyu101, Pocket-size, *C. moschata*, Yuemei No. 1, Wushan, Super long pumpkin and Xiangyu2, had disease incidences ranging from 60.46 to 77.50% and were susceptible to strain RS378. Eight *C. moschata* cultivars, including Butternut squash, Jingou, Zhongnan, Jinling, Xiangmi-Xiangyu, Pumpkin King, Jinniu No. 3 and No. 3 had disease incidences that ranged from 81.50 to 94.92% and were highly susceptible to strain RS378. Four *C. maxima* cultivars, including Black rose 879, Longxindan, Jinhongxi889 and Red earth, had disease incidences ranging from 83.62 to 93.73% and were highly susceptible to strain RS378 (Table 3).

**Table 3:** Pathogenicity of strain RS378 of *R. solanacearum* on cultivars of *C. maxima* and *C. moschata*.

**Varieties**	**Cultivars**	**Average disease incidence (%) ± SD value**	**RR^a^**
*C. maxima*	Black rose 879	83.62 ± 9.95	HS^b^
	Longxindan	88.08 ± 9.01	HS
	Jinhongxi889	89.34 ± 5.23	HS
	Red earth	93.73 ± 5.69	HS
*C. moschata*	Xiangyu1	38.63 ± 13.43	R
	Xiangmi	57.88 ± 8.34	MR
	Xiangyu101	60.46 ± 9.44	S
	Pocket-size	60.94 ± 5.41	S
	*C. moschata*	65.33 ± 3.07	S
	Yuemei NO.1	71.98 ± 11.02	S
	Wushan	75.48 ± 11.29	S
	Super long pumpkin	75.49 ± 2.31	S
	Xiangyu2	77.50 ± 13.08	S
	Butternut squash	81.50 ± 11.90	HS
	Jingou	81.88 ± 3.92	HS
	Zhongnan	85.89 ± 6.42	HS
	Jinling	87.84 ± 5.50	HS
	Xiangmi-Xiangyu	88.34 ± 3.51	HS
	Pumpkin King	91.76 ± 1.87	HS
	Jinniu NO.3	94.35 ± 3.24	HS
	NO.3	94.92 ± 1.60	HS

a *Disease incidence (DI) of diseased plants were assessed at 5 weeks after inoculation, calculated as DI = 100 × number of disease plants/ total number inoculated plants of each plot experiment (30)*.

b *Resistance reaction (RR) of 21 cultivars to strain RS378 was evaluated based on DI, where HR, highly resistant; R, resistant; MR, moderately resistant; S, susceptible; and HS, highly susceptible*.

## Discussion

A newly observed bacterial wilt disease in *C. maxima* in China is caused by *R. solanacearum*. In this study, a total of 24 isolates were obtained from *C. maxima* plants with bacterial wilt in Guangdong province, China, in 2016. These isolates produced typical *R. solanacearum*-type colonies on TZC medium plates (Figure 2). The colony characteristics of these isolates were consistent with previous descriptions of *R. solanacearum* (Kelman, 1954; She et al., 2012). All the isolates were pathogenic in *C. maxima* after artificial inoculation in the greenhouse, and they caused wilt symptoms similar to those observed in the field (Figure 1). The same pathogens were re-isolated from the inoculated diseased plants. The 16S rRNA gene sequences from these isolates, which were identical to each other, shared 100% identity with the *R. solanacearum* strain GMI1000 sequence from the GenBank database. According to the sizes of Pmx- and phylotype I-specific PCR products, these isolates were also identified to be *R. solanacearum*.

All of the *R. solanacearum* isolates from *C. maxima* in this study produced typical *R. solanacearum*-type colonies that were irregular, round, fluidal, and white with a pink center on TZC medium plates (Figure 2). However, eight isolates secreted a brown pigment (Figure 2B). According to their carbohydrate utilization patterns, all of the isolates that produced brown pigment were classified as biovar 4. Interestingly, we also isolated seven brown pigment-producing strains from *Z. officinale* that also belong to biovar 4 (unpublished data). The purpose of brown pigment production by *R. solanacearum* is unclear. Previous studies of *R. solanacearum* biovars from other hosts have shown that biovars 3 and 4 are dominant biovars in Guangdong (Zeng and Dong, 1995), consistent with this study. Biovars 3 and 4 were isolated from *C. maxima*, while biovars 1, 2, and 5 were not.

Virulence differentiation of *R. solanacearum* from *C. maxima* in different hosts was observed in this study. All of the isolates from *C. maxima* were highly virulent to *S. lycopersicum* and *S. melongena*, weakly virulent to *C. annuum* and *N. tabacum* and avirulent to *M. nana*. Six of eight biovar 3 isolates were weakly virulent to *Z. officinale*, whereas 16 biovar 4 isolates and two biovar 3 isolates, RS362 and RS366 were avirulent to *Z. officinale*. Based on their wide host range that includes four solanaceous crops, *S. lycopersicum, S. melongena, C. annuum*, and *N. tabacum* (Table 2), the twenty-four isolates were all classified into race 1, indicating they are quite different from a strain that was isolated from *C. maxima* in Colombia, which was classified as race 2 (Romo et al., 2012). The Pmx- and phylotype I-specific PCR and phylogenetic analysis (Figures 3, 4) results illustrated that the 24 isolates belong to phylotype I. Thus, the *R. solanacearum* isolates from *C. maxima* in this study belong to phylotype I, race 1, and biovar 3 or 4; this finding is consistent with the latest hierarchical classification scheme proposed by Fegan and Prior (2005) in which all *R. solanacearum* isolates from Asia are phylotype I (race 1: bv 3, 4, and 5).

Phylotype I is the lineage with the highest evolutionary divergence among the four phylotypes of *R. solanacearum*. Phylogenetic analysis of the *egl* and *hrpB* genes revealed that the 24 isolates of *R. solanacearum* from *C. maxima* clustered into three sequevars, including sequevars 17, 45, and 56 (Figure 3). Among these three sequevars, only sequevar 17 has been reported to infect *S. lycopersicum, S. melongena, C. annuum, N. tabacum, Solanum tuberosum*, and *Pogostemon cablin* in China (Xu et al., 2009; She et al., 2012). Sequevar 45 has not been previously described in China. Sequevar 56, the isolates of which clustered in a unique clade based on phylogenetic analysis of the *egl* gene, is a novel sequevar (Figure 3). Novel sequevars may result from the continuous evolution of phylotype I strains, which enables them to develop a wide host range and to adapt to extreme environments, providing virulence plasticity (Liu et al., 2017).

Planting resistant cultivars is effective and the simplest method for controlling bacterial wilt (Wang et al., 1998). In this study, variation analysis of the resistance of pumpkin cultivars to *R. solanacearum* strain RS378 showed that two *C. moschata* cultivars, Xiangyu1 and Xiangmi, were resistant or moderately resistant to strain RS378, but the 15 other *C. moschata* cultivars were highly susceptible or susceptible. Four *C. maxima* cultivars were all highly susceptible to strain RS378. Because of the limited number of cultivars of *C. maxima* used in this study, determining whether there are cultivars of *C. maxima* that are resistant to *R. solanacearum* is difficult. More pumpkin varieties must be collected and further evaluated for *R. solanacearum* resistance.

*Ralstonia solanacearum* is pathogenic on more than 200 plant species belonging to over 50 botanical families worldwide (Genin and Denny, 2012), and more host plants will be identified through surveys of *R. solanacearum*. In Guangdong, where temperatures are high (over 30°C) for approximately half the year, serious bacterial wilt disease often occurs. During the past ten years, seven new host plants, *Ipomoea aquatic, Canna generalis, Hedychium coronarium, Zinnia elegans, Solidago canadensis, Pogostemon cablin*, and *Ageratum conyzoides*, have been reported to be infected by *R. solanacearum* in Guangdong, China (He et al., 2008; Liu et al., 2009; She et al., 2012, 2013). In this paper, we report that *R. solanacearum* race 1 can infect *C. maxima* plants and cause wilt disease. Romo et al. (2012) reported that a *R. solanacearum* strain belonging to race 2 was isolated from *C. maxima* in Colombia, but we could not obtain further information on the biovar, sequevar, or phylotype of the strain or its pathogenicity and virulence attributes in *C. maxima* or other Solanaceae plants. To the best of our knowledge, this is the first report of bacterial wilt caused by *R. solanacearum* race 1 in *C. maxima* in the world.

## Conclusion

Bacterial wilt disease in *C. maxima* in Guangdong, China, is caused by *R. solanacearum*. All 24 strains isolated from *C. maxima* in this study belong to phylotype I, race 1 and biovar 3 or 4. These isolates clustered into sequevars 17, 45, and 56. Sequevar 45 was identified in China for the first time in this study, and sequevar 56 is a novel sequevar that has not been described previously. This study is the first report to identify *R. solanacearum* race 1 infections in *C. maxima*. Two *C. moschata* cultivars, Xiangyu1 and Xiangmi, were resistant or moderately resistant to *R. solanacearum* strain RS378. Our results provide valuable information for the further development of control strategies for *C. maxima* wilt disease.

## Author contributions

All authors made a substantial, direct and intellectual contribution to the work; XS wrote the original draft, and ZH revised the manuscript.

### Conflict of interest statement

The authors declare that the research was conducted in the absence of any commercial or financial relationships that could be construed as a potential conflict of interest.

## References

[B1] Buddenhagen I. Sequeira L. Kelman A. (1962). Designation of races in *Pseudomonas solanacearum*. Phytopathology 52:726.

[B2] Castillo J. A. Greenberg J. T. (2007). Evolutionary dynamics of *Ralstonia solanacearum*. Appl. Enviro. Microbiol. 73, 1225–1238. 10.1128/AEM.01253-06 17189443 PMC1828673

[B3] Cook D. Barlow E. Sequeira L. (1989). Genetic diversity of *Pseudomonas solanacearum:* detection of restriction fragment length polymorphisms with DNA probes that specify virulence and the hypersensitive response. Mol. Plant. Micro. Int. 2, 113–121. 10.1094/MPMI-2-113

[B4] Cook D. Sequeira L. (1994). Strain differentiation of *Pseudomonas solanacearum* by molecular genetic methods, in Bacterial Wilt: The Disease and Its Causative Agent, Pseudomonas solanacearum, ed. Hayward A. C. Hartman G. L. (Wallingford, UK: CAB International l), 77–93.

[B5] Denny T. (2006). Plant pathogenic Ralstonia species, in Plant-Associated Bacteria, ed. Gnanamanickam S. S. (Dordrecht: Springer), 573–644.

[B6] Fegan M. Prior P. (2005). How complex is the *Ralstonia solanacearum* species complex, in Bacterial wilt disease and the Ralstonia solanacearum species complex, ed. Allen C. Prior P. Hayward A. C. (St. Paul, MN: APS), 449–462.

[B7] Fegan M. and Prior, P. (2006). Diverse members of the *Ralstonia solanacearum* species complex cause bacterial wilts of banana. Aust. Plant Pathol. 35, 93–101. 10.1071/AP05105

[B8] Fegan M. Taghavi M. Sly L. I. Hayward A. C. (1998). Phylogene, diversity and molecular diagnostics of *Ralstonia solanacearum*, in Bacterial Wilt Disease: Molecular and Ecological Aspects, eds Prior P. Allen C. Elphinstone J. (Berlin; Heidelberg: Springer), 19–33.

[B9] Gabriel M. S. T. Cellie G. Guilbaud C. Kahane R. Allen C. Prior P. (2009). Broad diversity of *Ralstonia solanacearum* strains in Cameroon. Plant Dis. 93, 1123–1130. 10.1094/PDIS-93-11-1123 30754584

[B10] Genin S. Denny T. P. (2012). Pathogenomics of the *Ralstonia Solanacearum* species complex. Ann. Rev. Phytopathol. 50, 67–89. 10.1146/annurev-phyto-081211-173000 22559068

[B11] Hall T. A. (1999). BioEdit: a user-friendly biological sequence alignment editor and analysis program for Windows 95/98/NT. Nucleic Acids Symp. Ser. 41, 95–98.

[B12] Hayward A. C. (1964). Characteristics of *Pseudomonas solanacearum*. J. Appl. Bacteriol. 27, 265–277. 10.1111/j.1365-2672.1964.tb04912.x

[B13] Hayward A. C. (1991). Biology and epidemiology of bacterial wilt caused by *Pseudomonas solanacearum*. Ann. Rev. Phytopathol. 29, 65–87. 10.1146/annurev.py.29.090191.000433 18479193

[B14] Hayward A. C. (1994). Systematics and phylogeny of Pseudomonas solanacearum and related bacteria, in Bacterial Wilt: The Disease and Its Causative Agent, Pseudomonas solanacearum, eds Hayward A. C. Hartman G. L. (Wallingford, UK: CAB International), 123–135.

[B15] He L. Y. (1985). Bacterial wilt in the People's Republic of China, in Bacterial Wilt Disease in Asia and the South Pacific, ed Perley G. J. (Canberra: Proceedings of an international workshop held at PCARRD), 40–48.

[B16] He L. Y. Sequeira L. Kelman A. (1983). Characteristics of strains of *Pseudomonas solanacearum*. Plant Dis. 67, 1357–1361. 10.1094/PD-67-1357

[B17] He Z. F. She X. M. Yu H. Luo F. F. LI H. P. (2008). Pathogen identification of bacterial wilt of *Ipomoea aquatic*. Acta Phytopathol. Sin. 38, 120–125. 10.3321/j.issn:0412-0914.2008.02.002

[B18] Kelman A. (1954). The relationship of pathogenicity in *Pseudomonas solanacearum* to colony appearance on a tetrazolium medium. Phytopathology 44, 693–695.

[B19] Li X. Dorsch M. Del Dot T. Sly L. I. Stackebrandt E. Hayward A. C. (1993). Phylogenetic studies of the rRNA group II pseudomonads based on 16S rRNA gene sequences. J. Appl. Bacteriol. 74, 324–329. 10.1111/j.1365-2672.1993.tb03032.x

[B20] Li Y. Y. Feng J. Liu H. L. Wang L. Hsiang T. Li X. H. (2016). Genetic diversity and pathogenicity of *Ralstonia solanacearum* causing tobacco bacterial wilt in China. Plant Dis. 100, 1288–1296. 10.1094/PDIS-04-15-0384-RE 30686189

[B21] Lin C. H. Tsai K. C. Prior P. Wang J. F. (2014). Phylogenetic relationships and population structure of *Ralstonia solanacearum* isolated from diverse origins in Taiwan. Plant Pathol. 63, 1395–1403. 10.1111/ppa.12209

[B22] Liu Q. G. Zeng W. D. Zheng X. H. Che Z. P. (2009). Identification of pathogenic bacteria from four species of flowering plants. J. Huazhong Agri. Univ. 28, 277–280. 10.3321/j.issn:1000-2421.2009.03.006

[B23] Liu Y. Wu D. S. Liu Q. P. Zhang S. T. Tang Y. M. Jiang G. F. (2017). The sequevar distribution of *Ralstonia solanacearum* in tobacco-growing zones of China is structured by elevation. Eur. J. Plant Pathol. 147, 541–551. 10.1007/s10658-016-1023-6

[B24] Ma Q. C. Gao Y. C. (1956). Wilt disease of peanut. Bull. Fujian Acad. Agric. Sci. 2, 89–98.

[B25] Opina N. Tavner F. Hollway G. Wang J. F. Li T. H. Maghirang R. (1997). A novel method for development of species and strain-specific DNA probes and PCR primers for identifying *Burkholderia solanacearum*. Asia Pacific J. Mol. Biol. Biotechnol. 5, 19–30.

[B26] Pegg K. G. Moffett M. (1971). Host range of the ginger strain of *Pseudomonas solanacearum* in Queensland. Aust. J. Exp. Agric. Anim. Hus. 11, 696–698. 10.1071/EA9710696

[B27] Poussier S. Prior P. Luisetti J. Hayward C. Fegan M. (2000b). Partial sequencing of the *hrpB* and endoglucanase genes confirms and expands the known diversity within the *Ralstonia solanacearum* species complex. Syst. Appl. Microbiol. 23, 479–486. 10.1016/S0723-2020(00)80021-1 11249017

[B28] Poussier S. Trigalet-Demery D. Vandewalle P. Goffinet B. Luisetti J. Trigalet A. (2000a). Genetic diversity of *Ralstonia solanacearum* as assessed by PCR-RFLP of the hrp gene region, AFLP and 16S rRNA sequence analysis and identification of an African subdivision. Microbiology 146, 1679–1692. 10.1099/00221287-146-7-1679 10878132

[B29] Prior P. Ailloud F. Dalsing L. B. Remenat B. Sanchez B. Allen C. (2016). Genomic and proteomic evidence supporting the division of the plant pathogen *Ralstonia solanacearum* into three species. BMC Genomics 17:90. 10.1186/s12864-016-2413-z 26830494 PMC4736150

[B30] Prior P. Fegan M. (2005). Recent development in the phylogeny and classification of *Ralstonia solanacearum*. Acta Hortic. 695, 127–136. 10.17660/ActaHortic.2005.695.14

[B31] Romo J. P. Osorio J. G. M. II Yepes M. S. (2012). Identification of new hosts for *Ralstonia solanacearum* (Smith) race 2 from Colombia. Rev. Proteccion Veg. 27, 51–161. Available online at: http://scielo.sld.cu/scielo.php?script=sci_arttext&pid=S1010-27522012000300003&lng=en&tlng=en

[B32] Safni I. Cleenwerck I. Vos D. P. Fegan M. Sly L. Kappler U. (2014). Polyphasic taxonomic revision of the *Ralstonia solanacearum* species complex: proposal to emend the descriptions of *Ralstonia solanacearum* and *Ralstonia syzygii* and reclassify current *R. syzygii* strains as *Ralstonia syzygii subsp. syzygii subsp*. nov., *R. solanacearum* phylotype IV strains as *Ralstonia syzygii subsp*. indonesiensis subsp. nov., banana blood disease bacterium strains as *Ralstonia syzygii* subsp. celebesensis subsp. nov. and *R. solanacearum* phylotype I and III strains as *Ralstonia pseudosolanacearum* sp. nov. Int. J. Syst. Evol. Microbiol. 64, 3087–3103. 10.1099/ijs.0.066712-0 24944341

[B33] She X. M. He Z. F. Luo F. F. (2012). Pathogen identification of *Pogostemon cablin* bacterial wilt in Guangdong. Acta Phytopathol. Sin. 42, 569–576. 10.13926/j.cnki.apps.2012.06.003

[B34] She X. M. He Z. F. Luo F. F. (2013). Pathogen identification of *Ageratum conyzoides* bacterial wilt disease and its biological characteristics. Acta Phytophyl. Sin. 40, 533–539. 10.13802/j.cnki.zwbhxb.2013.06.001

[B35] Taghavi M. Hayward A. C. Sly L. I. Fegan M. (1996). Analysis of the phylogenetic relationships of strains of *Burkholderia solanacearum, Pseudomonas syzygii*, and the Blood Disease Bacterium of banana based on 16S rRNA gene sequences. Int. J. Syst. Bacteriol. 46, 10–15. 10.1099/00207713-46-1-10 8573483

[B36] Tamura K. Stecher G. Peterson D. Filipski A. Kumar S. (2013). MEGA6: molecular evolutionary genetics analysis version 6.0. Mol. Biol. Evol. 30, 2725–2729. 10.1093/molbev/mst197 24132122 PMC3840312

[B37] Wang J. F. Hanson P. Barnes J. A. (1998). Worldwide Evaluation of an International set of resistance sources to bacterial wilt in tomato. in Bacterial Wilt Disease, ed. Prior P. Allen C. Elphinstone J. (Berlin; Heidelberg: Springer), 269–275.

[B38] Wang T. Lin W. Huang Y. L. Yuan G. Q. Li Q. Q. (2009). Identification of the pathogen of *Siraitia grosvenorii* wilt. Acta Phytopathol. Sin. 39, 318–320. 10.3321/j.issn:0412-0914.2009.03.016

[B39] Wicker E. Lefeuvre P. de Cambiaire J. C. Lemaire C. Poussier S. Prior P. (2012). Contrasting recombination patternas and demographic histories of the plant pathogen *Ralstonia solanacearum* inferred from MLSA. ISME J. 6, 961–974. 10.1038/ismej.2011.160 22094345 PMC3329105

[B40] Woese C. R. Gutell R. Noller H. F. (1983). Detailed analysis of the higher-order structure of 16S-like ribosomal ribonucleic acids. Microbiol. Rev. 47, 621–669. 6363901 10.1128/mr.47.4.621-669.1983 PMC283711

[B41] Xu J. Pan Z. C. Prior P. Xu J. Zhang Z. Zhang H. (2009). Genetic diversity of *Ralstonia solanacearum* strains from China. Eur. J. Plant Pathol. 125, 641–653. 10.1007/s10658-009-9512-5

[B42] Zeng X. M. Dong C. (1995). Biotypes of *Pseudomonas solanacearum* (Smith) Smith from various hosts in Guangdong province. J. South Chin. Agric. Univ. 67, 50–53.

[B43] Zheng G. B. Fan H. Z. (1962). Pathogen identification of sweet potao bacterial wilt disease. Acta Phytophyl. Sin. 1, 243–253.

